# Transforming Medical Education: Assessing the Integration of ChatGPT Into Faculty Workflows at a Caribbean Medical School

**DOI:** 10.7759/cureus.41399

**Published:** 2023-07-05

**Authors:** Joseph Cross, Raymond Robinson, Sumanth Devaraju, Andrea Vaughans, Ricardo Hood, Tarron Kayalackakom, Prasanna Honnavar, Sheetal Naik, Roopa Sebastian

**Affiliations:** 1 Biochemistry, Cell Biology & Genetics, American University of Antigua, St. John's, ATG; 2 Microbial Pathogenesis and Immunology, Texas A&M College of Medicine, College Station, USA; 3 Clinical Sciences, American University of Antigua, St. John's, ATG; 4 Family Medicine, American University of Antigua, St. John's, ATG; 5 Medical Education and Simulation, American University of Antigua, St. John's, ATG; 6 Microbiology, American University of Antigua, St. John's, ATG; 7 Physiology, American University of Antigua, St. John's, ATG

**Keywords:** virtual assistant, survey, medical faculty, artificial intelligence, chatgpt, medical education

## Abstract

Introduction: ChatGPT is a Large Language Model (LLM) which allows for natural language processing and interactions with users in a conversational style. Since its release in 2022, it has had a significant impact in many occupational fields, including medical education. We sought to gain insight into the extent and type of usage of ChatGPT at a Caribbean medical school, the American University of Antigua College of Medicine (AUA).

Methods: We administered a questionnaire to 87 full-time faculty at the school via email. We quantified and made graphical representations of the results via Qualtrics Experience Management software (QualtricsXM, Qualtrics, Provo, UT). Survey results were investigated using bar graph comparisons of absolute numbers and percentages for various categories related to ChatGPT usage, and descriptive statistics for Likert scale questions.

Results: We found an estimated 33% of faculty were currently using ChatGPT. There was broad acceptance of the program by those who were using it and most believed it should be an option for students. The primary task ChatGPT was being used for was multiple choice question (MCQ) generation. The primary concern faculty had was incorrect information being included in ChatGPT output.

Conclusion: ChatGPT has been quickly adopted by a subset of the college faculty, demonstrating its growing acceptance. Given the level of approval expressed about the program, we believe ChatGPT will continue to form an important and expanding part of faculty workflows at AUA and in medical education in general.

## Introduction

Large language models (LLMs) are a type of Artificial Intelligence (AI) that allow for natural language processing and are a neural network model, trained on internet data which can predict the next most likely word in a sentence. Recent LLM iterations have shown an ability to comprehend, abstract, and reason in an almost human-equivalent fashion [1]. ChatGPT is a conversational AI agent based on GPT-3.5, an LLM developed by OpenAI (San Francisco CA) which features 175 billion parameters and leverages extensive data from the internet [2]. Since its public release in November 2022, the model has been widely adopted across various domains, such as history, banking, and entertainment [3].

A research paper by Kung et al. revealed that ChatGPT version 3.5 (ChatGPT 3.5) managed to score at or near the pass mark of 60% on all three standardized tests for the United States Medical Licensing Examination (USMLE) [2]. The results of this study were even more remarkable as they were achieved without any specialized training of the LLM, which is known as a zero-shot prompting strategy. ChatGPT performed slightly better on the last two tests which have more open-ended question formats, than the first test which is multiple choice question (MCQ) based. More recently, Nori et al. used ChatGPT version 4 (ChatGPT4) to achieve a remarkable average of over 80% in a zero-shot test of the same exams [4].

Hosseini et al. recently published the results of a survey sent to participants in a hybrid discussion event on the use of ChatGPT in education, research, and healthcare [5]. They found the greatest uncertainty in the use of ChatGPT was in the context of education. Other papers relevant to medical education have included the use of ChatGPT in clinical vignette generation, radiology, medical research, and the ethics of AI use [6-9].

Impact Research has recently disclosed the findings of a survey on the use of ChatGPT among K12 teachers and students aged 12-17 [10]. This survey revealed most of the teachers surveyed were already using ChatGPT in their work and around a third of students were using it in some capacity. Overall, a large majority of students and teachers who had used ChatGPT felt it positively impacted education. 

The American University of Antigua College of Medicine (AUA) offers a four-year medical program and is situated in Antigua, West Indies. Students undertake two years of basic science studies on campus, followed by clinical rotations in the USA, Canada, or the UK. The school has a mix of MD and PhD-qualified faculty, with a relatively large cohort for whom English is not their first language.

Given the potential for ChatGPT to revolutionize many occupational areas, particularly medical education, we sought to obtain a broad snapshot of ChatGPT workflow integration by faculty at AUA. We asked whether ChatGPT was currently being used by AUA faculty, and if it was, how often, what tasks it was being used for, what problems the faculty encountered in using it, and what were their views on the use of ChatGPT at the college going forward.

## Materials and methods

To introduce ChatGPT to faculty at AUA, we conducted two seminars and a survey on the use of ChatGPT amongst faculty. The first seminar covered basic topics, including a brief description of LLMs, signing up for ChatGPT from OpenAI, recent papers relevant to medical education and ChatGPT, and examples of effective and ineffective prompts. The second seminar featured a panel of faculty who made presentations on the incorporation of ChatGPT into workflows and the advantages and disadvantages they encountered.

Survey administration was approved by the AUA Research Council, approval #2023-001. We conducted the survey using the Qualtrics Experience Management online platform (QualtricsXM, Qualtrics, Provo, UT). It featured non-compulsory questions, including satisfaction ratings for ChatGPT on a 5-point Likert scale, open and closed-ended questions on faculty use of ChatGPT (such as crafting clinical vignettes or building multiple-choice questions), and demographic data of the faculty (e.g., age and native language). QualtricsXM provides automatic graphical summaries of raw survey data, dependent on user requirements. We analyzed the survey results through bar graph representations, comparing absolute numbers and percentages across different categories pertaining to ChatGPT use. For questions based on the Likert scale, we used descriptive statistics.

## Results

**Figure 1:**
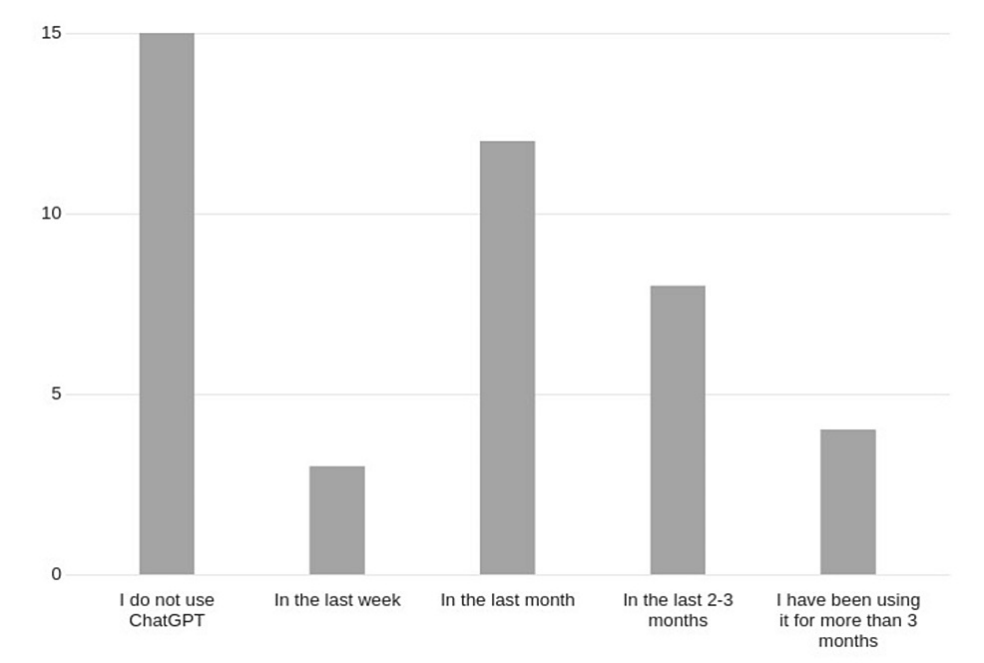
Faculty use of ChatGPT. Number of respondents for each category on the Y axis

Of the total 87 full-time faculty members, including participants from the Post Doctoral Teaching program, we received 44 responses. Of those who did respond to the survey, 29 (29/44 or 66%) were using ChatGPT, and the remainder of the respondents were non-users (Figure 1).

Assuming those among the faculty who did not respond at all to the survey were also non-users, we estimate the current usage among all faculty to be 33%. 

**Figure 2:**
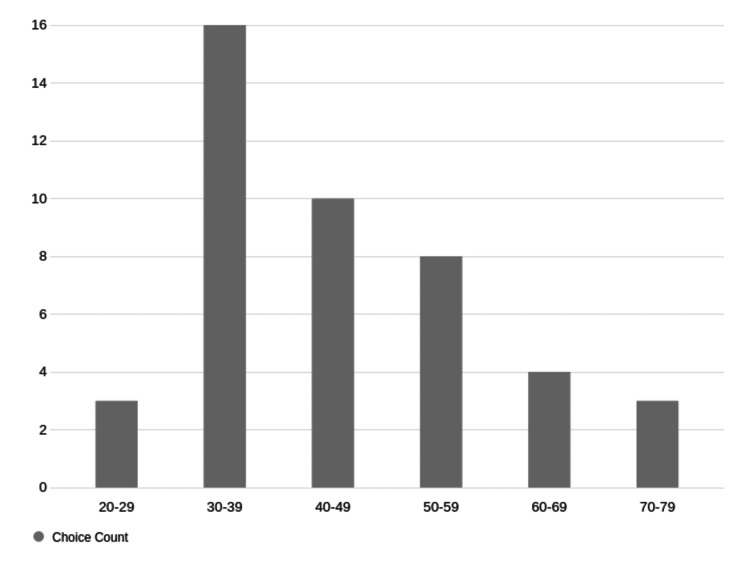
Faculty users of ChatGPT by age. Number of respondents for each category on the Y axis.

The majority of faculty using ChatGPT were in the age range 30-39, with a clear relationship between increasing age and decreasing usage of ChatGPT (Figure 2)

**Figure 3:**
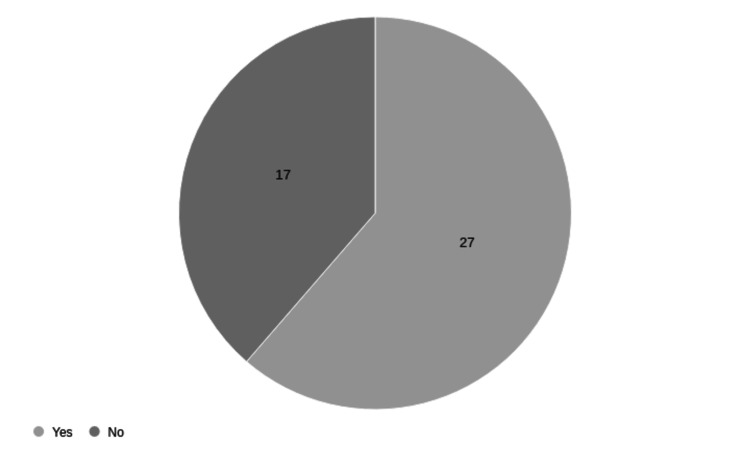
Faculty for whom English is their first language.

The demographic of faculty using ChatGPT for whom English is not the first language accurately mirrored the overall faculty composition (Figure 3).

**Figure 4:**
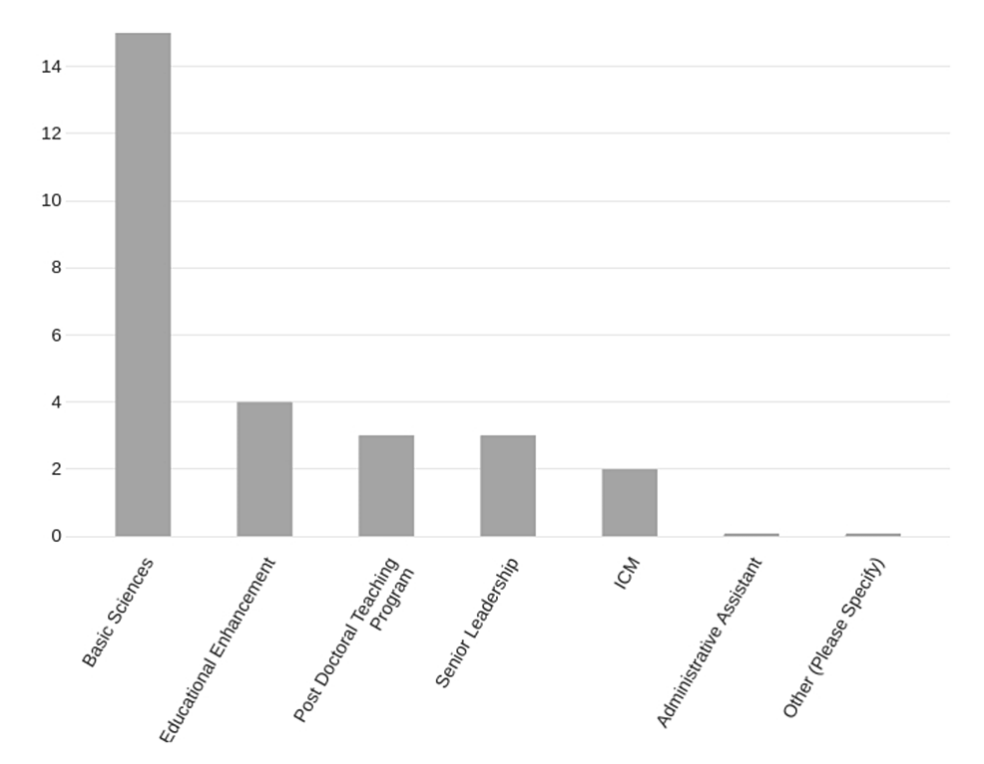
Faculty ChatGPT use by teaching area. Number of respondents for each category on the Y axis.

The majority of users were from the Basic Sciences Department, followed by the Education Enhancement Department (EED), maintaining a reflection of the usual proportions in those departments in many colleges (Figure 4).

**Figure 5:**
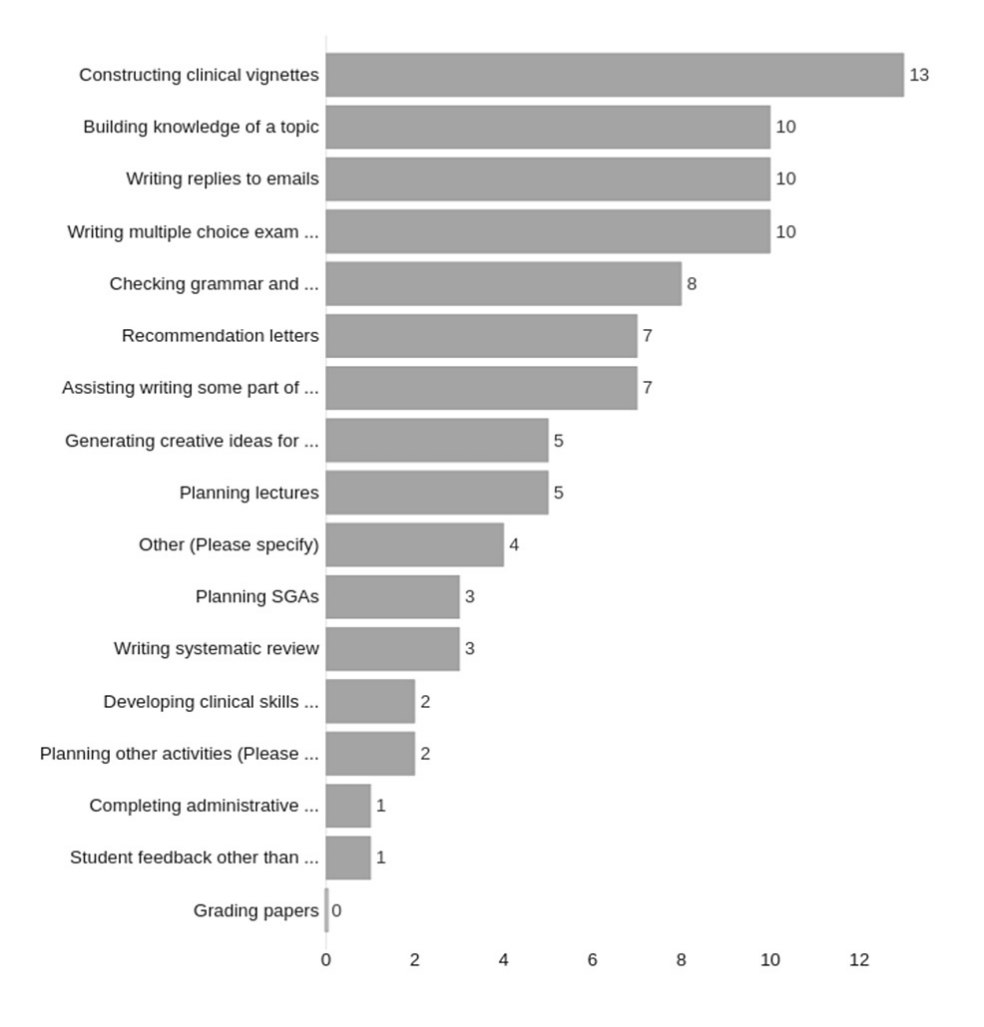
Faculty ChatGPT use by task. Number responses for each category on X axis. Individuals could respond to multiple categories.

A majority of faculty were using ChatGPT for clinical vignette construction (13/24 respondents, 54%) and the second highest usage was for MCQ construction, building knowledge of a topic or writing email replies (10/24, 42%) (Figure 5).

We consider clinical vignette and MCQ construction to be essentially part of the same task, as vignettes are part of the MCQ generation process. 

**Figure 6:**
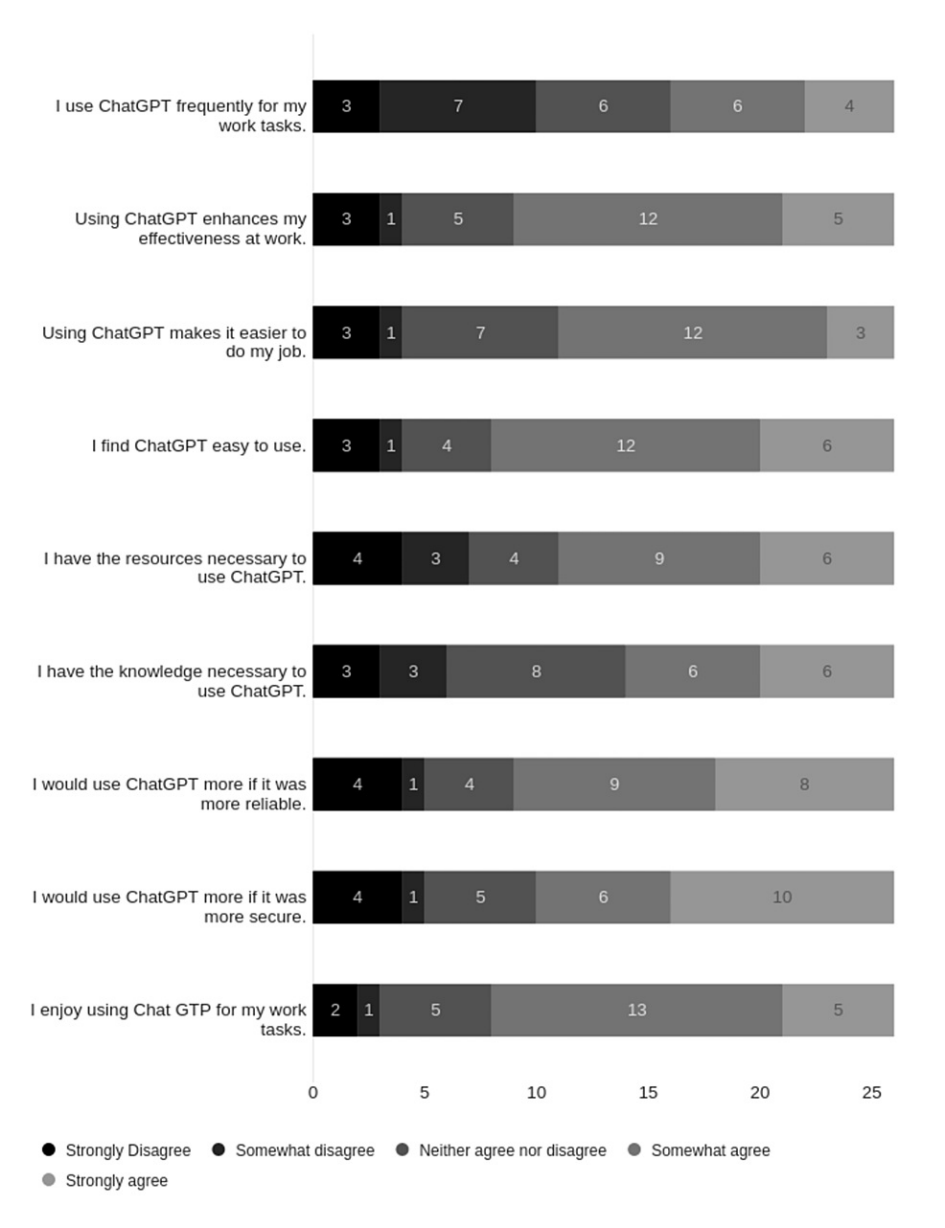
Likert scale of questions related to use of ChatGPT.

The overall impression from the Likert scale questions was that ChatGPT is viewed positively. The greatest level of agreement was for statements relating to ease of use and enjoyment of use (Figure 6).

**Figure 7:**
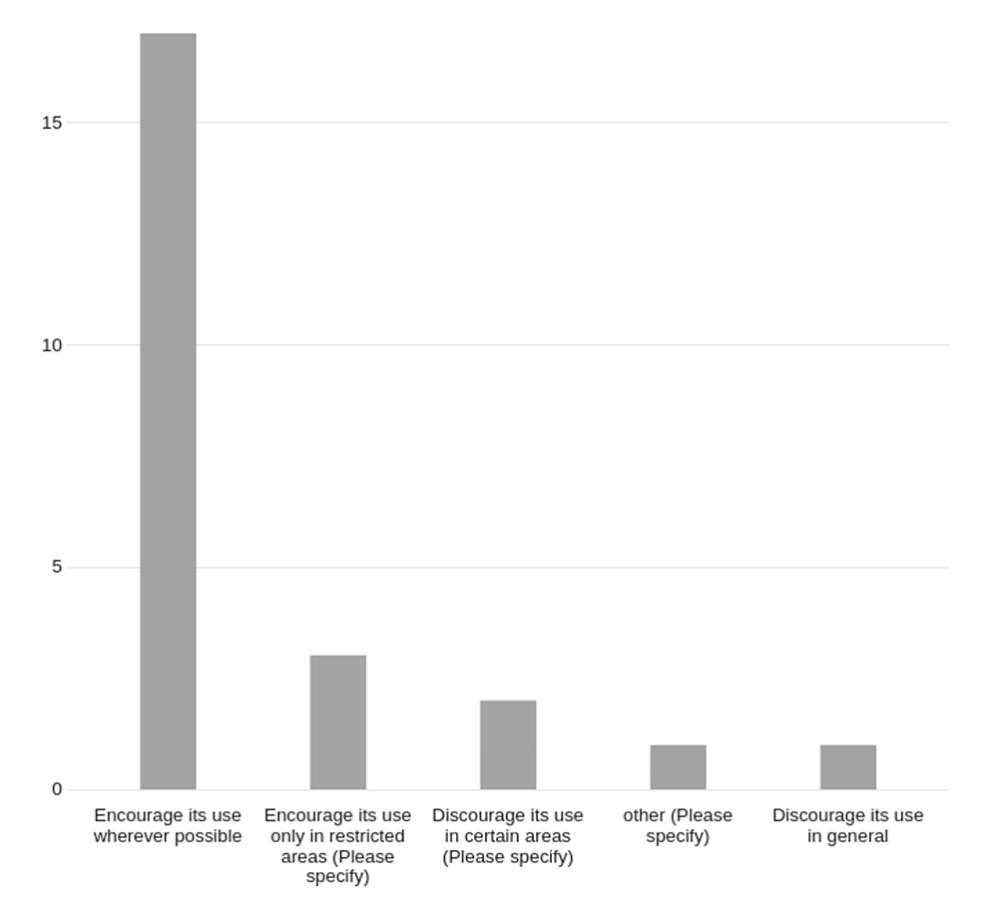
Faculty views on school policy for faculty ChatGPT use. Number of respondents for each category on the Y axis.

Most faculty believed ChatGPT should be optional for all faculty (17/24, 71%) (Figure 7).

**Figure 8:**
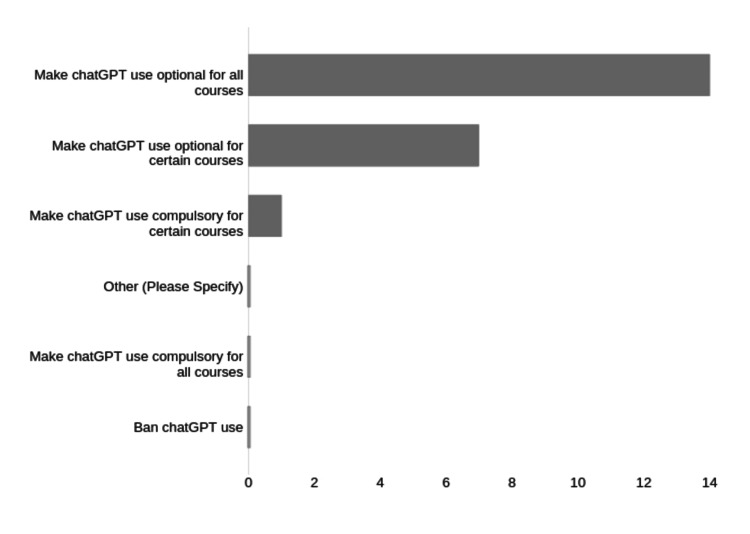
Faculty views on school policy for student use of ChatGPT. Number of respondents for each category on the X axis.

Most faculty also believed ChatGPT should be an option for students (14/22, 64%), with no faculty agreeing it should be banned (Figure 8).

**Figure 9:**
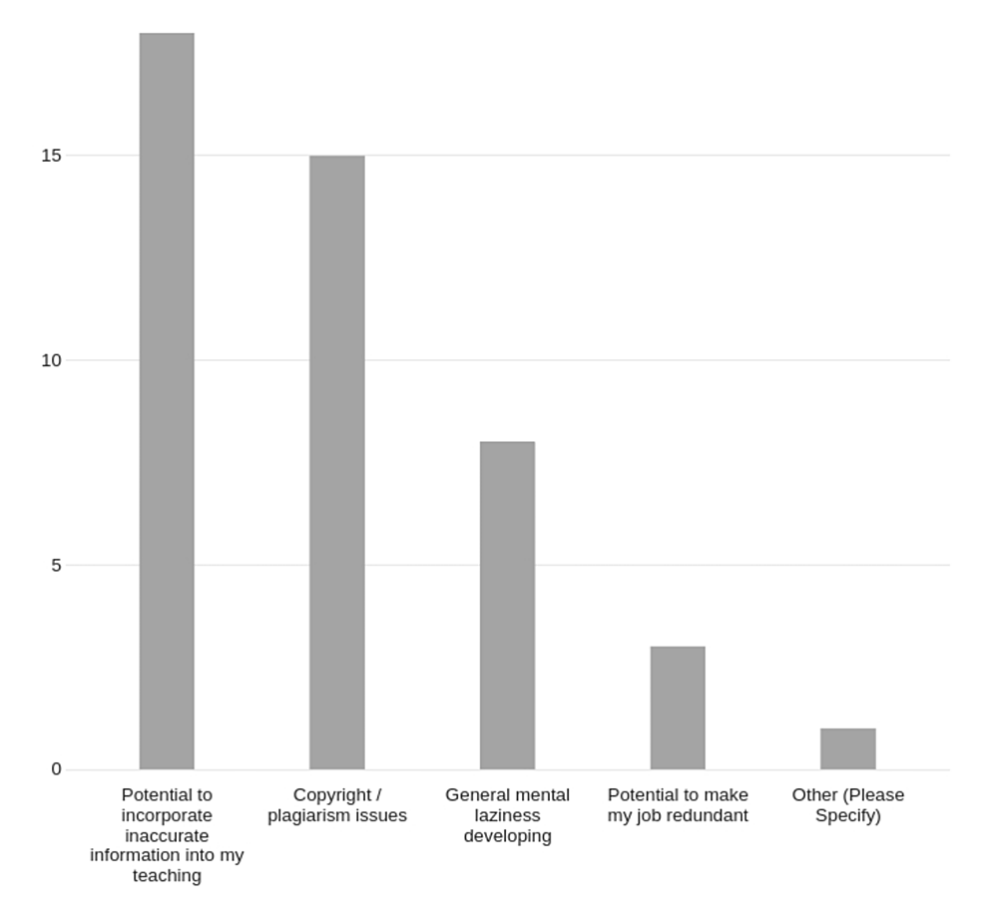
Faculty concerns around ChatGPT in medical education. Number of respondents for each category on the Y axis.

The major concern related to ChatGPT use was the incorporation of misinformation into teaching materials (17/23, 74%), and copyright/plagiarism was the second highest concern (15/23, 65%) (Figure 9).

## Discussion

We explored the uptake and usage of ChatGPT at a four-year medical school in the Caribbean. We administered an email survey to the 87 full-time faculty members on campus. Our data indicate that approximately one-third of them are currently using ChatGPT for their professional activities

Faculty in the younger age brackets were more likely users of ChatGPT, perhaps reflecting a greater familiarity with technology in general in younger users. Users for whom English was not the first language were broadly reflective of the proportions found among faculty overall.

MCQ construction (including clinical vignettes) was the most common way ChatGPT was used. We note that the school has recently introduced low-stakes summative quizzes based on MCQs written by the faculty themselves, rather than sourced externally. Summative question construction has been a time-consuming and ongoing task for most faculty and may have influenced the number of faculty who reported using ChatGPT for this purpose. Whether this will continue to be a large fraction of ChatGPT usage will depend on whether our question bank becomes sufficient and if no further questions are needed.

Surprisingly, building knowledge of a topic was also an important use for a relatively large number of respondents. It appears faculty are already trusting the information that is being generated from ChatGPT and using it as a “one-stop shop” for knowledge sourcing, rather than perhaps multiple sources spread over multiple modalities, as has been traditional.

We speculate that the type of tasks ChatGPT will be used for will change over time as priorities at the school change and as faculty experiment with different uses for the program. Usage patterns may also be dependent on whether institutional pricing becomes available through OpenAI, as has been predicted, which would then mean all faculty would have free professional access to ChatGPT4 and later versions as they become available [11]

There was a clear majority who believed school policy should encourage faculty ChatGPT use wherever possible. There was also a corresponding clear majority who believed ChatGPT use should be an option for students wherever possible, with no responses in favor of banning its use for students. This suggests a general trust and acceptance of ChatGPT, at least among those responding to the survey.

We speculate that the apparent rapid acceptance by a section faculty may be partly due to the school’s recent experience of switching from live to virtual classes during the COVID-19 pandemic. Most faculty anecdotally found this transition to have both positive and negative aspects. On the positive side, virtual meeting attendance was seen as much more efficient, as evidenced by the fact we have maintained this procedure at the school, even though virtual teaching is no longer conducted. On the negative side, face-to-face teaching was seen as more effective than virtual teaching. The school administration acknowledged this with a swift return to campus for students and faculty when the pandemic began to wane. Dunn et al. drew similar conclusions from a study of Emergency Medical educators [12]. It is possible many faculty now felt confident they could incorporate another electronic program or app into their teaching, having just had a successful experience doing something very similar.

The greatest faculty concern about ChatGPT use was its potential to generate incorrect information. Educational institutions are built on a foundation of trust between students and teachers that what is being taught is relevant and factually correct. The phenomenon of “hallucinating” information and references has now been well documented [13]. The fact that our faculty are finding filtering out incorrect information a problem suggests this is also an issue at AUA. This result is also supported by the results of Hosseini et al., although we note the occupational categories of respondents in their study do not match those in our study exactly [5]. These results do not match those of Sallam et al., who found ethical issues such as bias and plagiarism to be the main concern, followed by incorrect information generation as the second highest concern, in a systematic review on the utility of ChatGPT in the healthcare sector [14]. We found copyright/plagiarism, broadly equivalent to the ethics category of Sallam et al., to rank second on the list of concerns at AUA. We noted that the results of Sallam et al. included those from researchers and practicing physicians, as well as medical educators. We speculate that concerns around plagiarism would be particularly prevalent where research was the primary focus. We may expect a different prioritization of concerns at AUA, where education, not research, is the main focus.

Overall, in our experience, ChatGPT4 is superior to ChatGPT3.5 in terms of the veracity of information returned, although it does still occasionally return incorrect information. We speculate that as more faculty begin to use later versions, the issue of incorrect information being returned by ChatGPT will rank lower as an issue.

Our study was limited by the fact that we were unable to obtain a definitive picture of the extent of usage among faculty overall, as not all faculty completed the questionnaire. Low sample size also hampered our ability to conduct standard statistical tests of significance and therefore to draw firm statistical conclusions.

We will monitor ChatGPT uptake and usage by means of another questionnaire in six months. This will seek to determine whether initial/uptake has been maintained, whether the same pattern of tasks ChatGPT is used for has been maintained, whether unforeseen problems have emerged, and whether new unpredicted uses of ChatGPT have been discovered. In relation to students, we will also be administering a questionnaire on the impact on their study habits and performance. Presently, this is low anecdotally at the school, but we expect this to change rapidly.

## Conclusions

This study indicates a rapid uptake of ChatGPT by a relatively large proportion of faculty at a Caribbean medical school. Most faculty found that ChatGPT was easy and pleasurable to use, suggesting that the use will be ongoing and possibly extend to more faculty in the future. Currently, the major task that the faculty are using ChatGPT for is USMLE-style MCQ generation. The primary concern of faculty regarding ChatGPT is the potential for incorrect information to be returned and presumably incorporated into learning materials. There was a broad acceptance by study participants that ChatGPT should be integrated into the workflows of both faculty and students.
